# Diagnosis and treatment of uveitis; not restricted to the ophthalmologist

**Published:** 2015-09-30

**Authors:** Jan A. M. van Laar, Aniki Rothova, Tom Missotten, Robert W. A. M. Kuijpers, P. Martin van Hagen, Mirjam E. J. van Velthoven

**Affiliations:** 1 Department of Internal Medicine and Immunology, Clinical Immunology, Erasmus Medical Center, Rotterdam, The Netherlands; 2 Department of Ophthalmology, Erasmus Medical Center, Rotterdam, The Netherlands; 3 Uveitis Service, Rotterdam Eye Hospital, Rotterdam, The Netherlands

**Keywords:** uveitis, ethiology, anti-TNF, immunosuppressive therapy, multidisciplinary management

## Abstract

Uveitis is associated with a wide range of underlying causes. Familiarity with its clinical manifestations, referral indications, and treatment strategies are required for the optimal use of current therapeutic options. Uveitis can be caused by infectious and non-infectious factors, resulting in differing prognoses and treatments. The treatment of chronic, non-infectious uveitis has profoundly changed in the last years due to the advent of biologicals, but also of intraocular therapies. In severe uveitis, treatment of the underlying cause, whether ocular or systemic, is required to prevent severe loss of vision. For these purposes, a multidisciplinary clinical approach is important, which is addressed in this review.

**Relevance for patients**: A broad understanding of the different causes of uveitis and the implementation of disease-tailored, multidisciplinary management of uveitis is expected to improve treatment outcomes for patients with different types of uveitis.

**This article is adapted and translated with permission from: Ned Tijdschr Geneeskd. 2013; 157(38):A5703.**

**Edited by:** Marc De Smet, Specialized Eye Center for Uveitis and Retinal Diseases, Lausanne, Switzerland

## 1. Introduction

Patients with uveitis often present with nonspecific symptoms, ranging from blurry vision or a red eye to headaches and severe photophobia. Uveitis should be considered in patients who retain a low visual acuity despite adequate refractive correction with glasses. Due to the diverse etiology and presentation, patients with uveitis can be first encountered by a variety of medical professionals, other than ophthalmologists, including general practitioners, rheumatologists, pulmonologists, and internal medicine specialists. Uveitis should therefore be considered part of the differential diagnosis when a patient is first seen in any of these specialties.

Literally, uveitis refers to inflammation of the uveal tract (consisting of the iris, ciliary body, and choroid) of the eye.

However, all ocular structures, including the retina, vitreous body, and optic nerve can be implicated in the inflammatory process. Uveitis is classified according to the anatomical location of the disease: anterior uveitis (front of the eye), intermediate uveitis (middle of the eye), posterior uveitis (back of the eye), or panuveitis (involvement of the whole eye). The cause of uveitis can often be deduced from its anatomical location (Table 1) [1]. Uveitis is associated with a systemic disease in approximately 30% of cases. Another 30% is caused by an infectious agent, whereas the condition remains idiopathic in the remaining 40% of cases. It is crucial to rapidly differentiate between infectious and non-infectious uveitis inasmuch as the treatment and prognosis markedly differ. It should also be noted that the cause of uveitis differs per race, region, and socioeconomic status [2].

Uveitis can result in irreversible visual impairment and constitutes the underlying cause in approximately 10% of all cases of blindness. Visual loss results directly from the inflammatory process, which can cause macular edema or chorioretinal scarring, or is induced by severe complications such as glaucoma or cataract [3]. The incidence of uveitis in the Western world is 52.4 per 100,000 and the prevalence is 115 per 100,000 [2]. The incidence correlates positively with age and affects women more frequently than men. The morbidity rate is often high due to the chronic course of the disease and the frequent exacerbations. Consequently, uveitis can have a severe impact on a person’s quality of life [4].

**Table 1.** Anatomical classification of uveitis and overview of typical underlying causes in immunocompetent patients.

Location	Involved ocular structures	Non-infectious cause	Infectious cause
Anterior uveitis	- Iris - Ciliary body	- Behçet’s disease - Crohn’s disease - HLA-B27 associated - Juvenile idiopathic arthritis - Reiter’s disease - Sarcoidosis	- Cytomegalovirus - Herpes simplex virus - Mycobacterium tuberculosis - Rubella - Varicella zoster virus
Intermediate uveitis	- Vitreous - Peripheral retina	- Multiple sclerosis - Sarcoidosis	- Bartonella henselae - Borrelia burgdorferi
Posterior uveitis	- Retina - Choroid - Optic nerve	- Autoimmune disorders^*^ - Behçet’s disease - Birdshot chorioretinitis - Crohn’s disease - Sarcoidosis	- Herpes simplex virus - Mycobacterium tuberculosis - Toxoplasma gondii - Treponema pallidum - Varicella zoster virus - Borrelia burgdorferi
Panuveitis	- Entire inner eye	- Behçet’s disease - Sarcoidosis - Vogt-Koyanagi-Harada syndrome^#^	- Herpes simplex virus - Mycobacterium tuberculosis - Toxoplasma gondii - Treponema pallidum - Varicella zoster virus

* e.g., rheumatoid arthritis and granulomatosis with polyangiitis; more frequently associated with scleritis, but can also cause uveitis.

# Vogt-Koyanagi-Harada syndrome is a rare uveo-meningeal disorder characterized by sterile meningitis, uveitis, auditory symptoms, and vitiligo.

Physicians should be particularly vigilant for uveitis in patients with eye complaints that are concurrently at risk for toxoplasmosis, herpes, tuberculosis, or syphilis, as these are the most prevalent infectious causes of uveitis. Uveitis should also be part of the differential diagnosis in patients with ocular symptoms that concomitantly suffer from a systemic disorder or exhibit signs of a systemic disorder, such as arthritis, aphthous stomatitis, bowel complaints, or erythema nodosum. Referral to an ophthalmologist is indicated in these situations.

## 2. Infectious uveitis

Infectious uveitis can be caused by the intraocular presence of microorganisms, an immunological reaction to microorganisms, or a combination of both (Table 1). Blood tests are of limited diagnostic value because ocular involvement often occurs in the chronic phase of a systemic infection, at which point serology generally yields inadequate results. Another option to render a diagnosis is an anterior chamber tap, through which aqueous humor is obtained for analysis for the presence of microorganisms by PCR. In addition, this also enables the comparison of locally produced antibodies against microorganisms to systemic antibody levels, the so-called Goldmann-Witmer coefficient [5,6].

**Figure 1.**
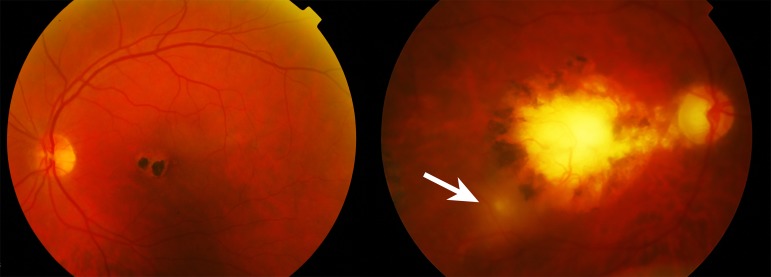
Color fundus photograph of chorioretinitis due to a toxoplasmosis infection. The left panel presents the left eye showing a central scar in the macula with both hypo- and hyperpigmentation. The right panel depicts exacerbation of active inflammation (hazy yellowish area at the edge of the pigmented scar (arrow)). The haziness of the right panel is attributed to inflammation of the vitreous.

The most common cause of infectious uveitis in Western countries such as the Netherlands is toxoplasmosis, which is characterized by focal chorioretinitis that is often associated with old satellite scars (Figure 1). Herpes viruses, such as the herpes simplex or varicella zoster virus, are also known to cause uveitis. These viruses mainly induce recurring anterior uveitis, with or without keratitis, or severe and destructive acute retinal necrosis. The latter frequently results in permanent vision loss or blindness, even when treated timely with antivirals [7]. Cytomegalovirus is typically seen in HIV-positive and transplant patients, but can also surface in immunocompetent subjects [8].

A less prevalent cause of uveitis is tuberculosis, although the incidence is increasing due to current immigration patterns and increased traveling. The recent introduction of interferon-γ release assays (e.g., Quantiferon or T-spot) has revealed that latent tuberculosis is found more frequently in otherwise unexplained uveitis [9,10]. There is, however, currently no validated test available to diagnose true ocular tuberculosis, also because detection of mycobacteria in ocular fluids or tissue is difficult [9,11-13].

Ocular manifestations are also seen in patients with syphilis and can be very diverse. It is imperative to always exclude this pathogen in patients with confirmed uveitis before immunosuppressant systemic therapy is started. Lyme’s disease can involve the eye in all stages, but uveitis typically arises in the chronic stage of the disorder. The typical clinical entity of heterochromic iridocyclitis (also known as Fuchs uveitis syndrome) is predominantly triggered by the rubella virus, particularly in Europe. It can lead to glaucoma and cataract and is mainly observed in unvaccinated patients [14,15]. Less frequent manifestations of uveitis are intraocular infections with candida or Aspergillus, which can arise in patients with a compromised immune system.

### 2.1. Treatment

In general, ocular infections should be considered a neurological manifestation of the underlying microbial infection, and must be treated accordingly. Additional glucocorticoid therapy can be considered in cases where the eye or visual acuity is at risk due to the concomitant local inflammatory response.

In the acute phase, toxoplasmosis is usually treated with a combination of antibiotics [16]. Superficial herpetic infections can be treated with topical antiviral ointment or with oral antivirals. Severe acute retinal necrosis, however, requires swift initiation of intravenous antivirals, although is some cases oral treatment is sufficient [17,18]. There is currently no effective treatment for latent toxoplasmosis or latent herpetic infections (e.g., herpes simplex, varicella zoster), which means that these patients suffer from recurrent episodes of uveitis.

Ocular tuberculosis is frequently confined to the eye and generally responds well to tuberculostatic treatment [9-13]. Whether or not treatment with tuberculostatics is required depends on the severity of the ophthalmological presentation, but is indicated in cases with a positive aqueous humor culture or PCR-confirmed ocular tuberculosis.

Long-term treatment is the best option in patients with frequently recurring uveitis or with impending vision loss. Multi-disciplinary management, involving ophthalmologists, infectious disease experts, microbiologists, and immunologists is mandatory for these cases.

## 3. Non-infectious uveitis

**Figure 2.**
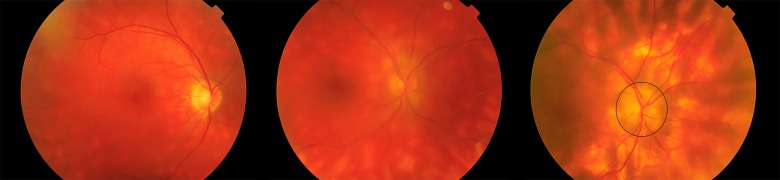
Color fundus photographs of the right eye of different patients with ‘Birdshot’ chorioretinitis, showing the characteristic multiple, yellow chorioretinal inflammatory lesions with increasing severity (from left to right). The right panel also shows mild swelling of the optic disc (encircled).

Non-infectious uveitis can be the first sign of an underlying systemic disorder (Table 1) or can be confined to the ocular structures, as is the case in ‘Birdshot’ chorioretinitis (Figure 2) and idiopathic uveitis (20-30% of all cases). Ophthalmologists frequently recognize the underlying cause of uveitis based on specific fundoscopic characteristics, its clinical presentation, or through initial laboratory results. Anterior uveitis is the most prevalent presentation of non-infectious uveitis and, in approximately half of cases, it is associated with the presence of the human leukocyte antigen B27 (HLA-B27) or its associated diseases. These patients typically suffer from severe recurring and alternating unilateral anterior uveitis and is most frequently encountered in males with ankylosing spondyloarthritis.

**Figure 3.**
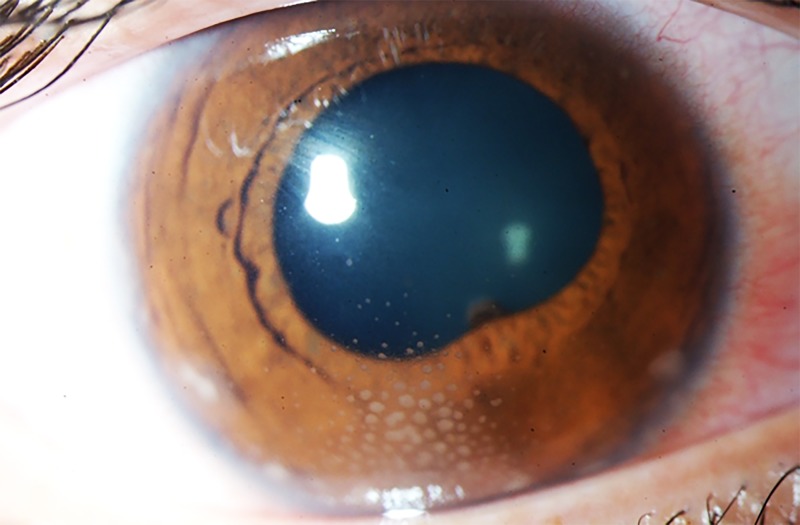
Color image of the anterior segment of the right eye of a patient with granulomatous anterior uveitis. Typical for this presentation are the mutton-fat keratic precipitates organized in ‘Arlt’s triangle’ (approximately 6 o’clock) on the interior surface of the cornea (endothelium). The image additionally shows mild ciliary hyperemia of the conjunctiva and adhesion of the iris to the lens capsule (pupil margin at approximately 5 o’clock).

**Figure 4.**
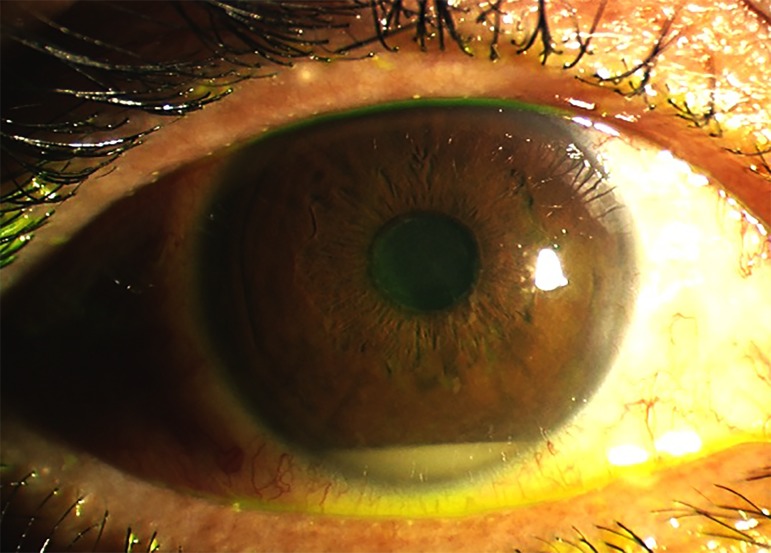
Color of the anterior segment of a patient with Behçet’s disease. Accumulation of granulocytes creating a liquid level in the anterior chamber is clearly visible. Although hypopyon is an archetypal ocular feature of Behçet’s disease, the visual prognosis is determined by changes in the posterior eye segment, including injury to the macula and optic nerve.

If slit lamp examination reveals the presence of intraocular granulomas (Figure 3) or periphlebitis, sarcoidosis should be considered. Sarcoidosis can affect all ocular structures and hence result in granulomatous anterior uveitis, posterior uveitis with occlusive vasculitis, retinal or vitreous hemorrhages or lacrimal gland, or orbital granuloma. Behçet’s disease is mainly seen in the Middle-East and Mediterranean population. This autoimmune vasculitis is characterized by aphthous stomatitis and genital ulcers. The ocular manifestations in Behçet’s disease are often severe and irreversible, ranging from anterior uveitis with hypopyon (Figure 4) to inflammatory retinal lesions with (occlusive) vasculitis and neuritis in the posterior segment of the eye that can acutely threaten the ability to see.

A number of multiple sclerosis (MS) patients experience ophthalmologic sequelae, ranging from optic neuritis to eye movement disorders and various forms of uveitis, which can even be the initial manifestation of MS. The most renowned ocular manifestation of MS is optic neuritis, albeit intermediate uveitis is also highly associated with MS [19]. The incidence of uveitis in MS is, however, less than 1%, while its prevalence ranges from 0-9.3% [20]. Various studies have found that uveitis precedes MS in almost 50% of cases [21]. Another neurological disorder linked to uveitis is Vogt-Koyanagi-Harada syndrome, which initially presents with severe headache and meningismus, followed by vitiligo and hair depigmentation.

In contrast to the above mentioned conditions, systemic disorders such as rheumatoid arthritis, granulomatosis with polyangiitis, relapsing polychondritis, and systemic lupus erythematosus are more likely associated with scleritis and episcleritis rather than uveitis. The masquerade syndrome is a rare situation in which malignant (hematological) cells are found inside the eye. It usually concerns B-cell lymphoma and can be associated with central nervous system involvement. This syndrome can be confused with uveitis, the main difference being that it responds poorly to conventional uveitis treatment. Even less prevalent are cases of carcinoma- or melanoma-related retinitis, which are mostly seen in conjunction with small-cell lung carcinoma or cutaneous melanoma.

### 3.1. Treatment

Immunosuppressant agents form the gold standard treatment for non-infectious uveitis. Most patients with non- infectious uveitis are treated with locally applied immunosuppressants. In anterior uveitis, topical steroids drops comprise the initial treatment, but are usually combined with cycloplegic drops for pain relief and prevention of posterior iris adhesions. In severe cases, periocular steroid injections are given.

**Figure 5.**
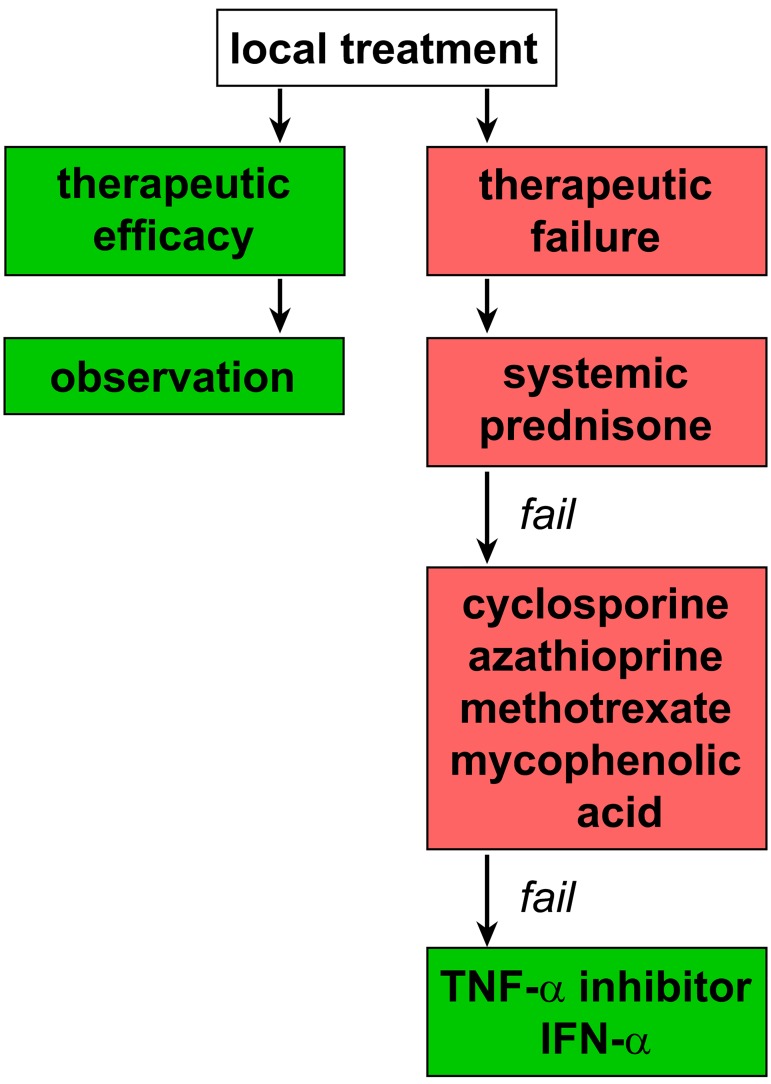
Flow diagram for the management of non-infectious uveitis.

If the clinical response is unsatisfactory in anterior uveitis, or when the posterior segment is mainly involved, or when there is severe bilateral involvement, patients are concurrently started on systemic glucocorticoids or ‘disease-modifying antirheumatic drugs’ (DMARDs, Figure 5). Although their mechanisms of action differ, all DMARDs can be used to treat non-infectious uveitis [22]. If vision is severely impaired, the initial treatment of choice is intravenous methylprednisolone (1 g, daily) for 1 to 5 days. Before the era of biologicals, intravenous cyclophosphamide could be used as an alternative for steroids in severe sight-threatening uveitis, although presently this treatment is reserved for refractive cases. Initial intravenous treatment is followed by maintenance therapy with oral prednisone and a DMARD. Before the introduction of effective immunosuppressive drugs, patients with e.g., Behçet’s disease inevitably became blind, which is still the case in regions that have limited access to immunomodulatory drugs. Treatment is generally continued up to 6 months after clinical remission. In cases of chronic refractory uveitis, it might even be necessary to continue maintenance therapy for several years.

Sarcoidosis is dealt with somewhat differently. The ophthalmologist will refer patients with signs and symptoms of sarcoidosis to another medical specialist in order to diagnose and subsequently treat the extra-ocular sarcoidosis. This is important as sarcoidosis can cause several severe health problems in absence of a clear clinical phenotype, including impaired lung function, increased liver enzymes (i.e., liver dysfunction caused by granulomas), hypercalcemia, or renal-, cerebral-, or cardiac manifestations.

The advent of biologicals has presented a new class of drugs that can be used to treat non-infectious uveitis [23]. The effective interferon alpha (IFN-α) was already used in the late 1990s to treat non-infectious uveitis. More recent clinical trials have evaluated the use of tumor necrosis factor alpha (TNF-α) inhibitors for the treatment of refractory uveitis [23-26]. Infliximab and adalimumab in particular are associated with an excellent and durable therapeutic efficacy in uveitis treatment. Anti-TNF therapy can be associated with mild infections such as viral upper respiratory tract infections, skin infections, and psoriasis-like skin lesions. It is also imperative to exclude the presence of MS, tuberculosis, and latent viral hepatitis before starting patients on TNF-α inhibitors. Infliximab is administered intravenously every two months and adalimumab is given subcutaneously every two weeks. It has to be taken into account that currently the use of biologicals is mostly “off-label,” pending FDA registration, and reimbursement settlements differ per country.

In cases of unilateral uveitis or when systemic medication is not well-tolerated, there are currently several options for intraocular treatment. Over the years several steroid implants have been introduced, which are composed of a slow-release system, enabling a therapeutic effect that lasts from 3 months to 3 years [27,28]. Another possibility, which is also used for the treatment of ocular lymphoma, is intravitreal injection of methotrexate.

## 4. Multi-disciplinary management of uveitis

### 4.1. Ophthalmological diagnostic screening

A Dutch national guideline for the diagnosis and treatment of uveitis has been formulated on the basis of the knowledge detailed above [29]. In clinical practice, the ophthalmologist usually initiates the first round of diagnostic screening, except in case of a first episode of mild anterior uveitis. Initial laboratory screening includes determination of serum C-reactive protein levels, circulating angiotensin-converting enzyme concentrations, erythrocyte sedimentation rate, a complete blood count, liver and kidney function tests, syphilis serology, and HLA-B27 genotyping (anterior uveitis only). Anti-nuclear antibodies (ANA) should be measured in children with anterior uveitis due to the close association with juvenile idiopathic arthritis. In case of scleritis rheumatic factor, ANA and anti-neutrophil cytoplasmic antibody are included in the analysis. Additional diagnostics such as a pulmonary CT scan, a brain MRI, HIV testing, or tuberculosis screening can be considered on a per-patient basis.

### 4.2. The role of other medical subspecialties

Following initial screening by an ophthalmologist, patients are referred to another medical specialist for further evaluation and to either treat the underlying cause of uveitis or assist in the initiation of the appropriate immunosuppressive or antibiotic treatment. The only exception to this rule is a first case of mild anterior uveitis, which warrants a less aggressive approach (Figure 5). The clinical presentation of the uveitis primarily determines the referral strategy. Patients that receive long-term immunosuppressive therapy are often monitored by internists or other medical subspecialists. Depending on the cause and severity of uveitis, the management of uveitis can be coordinated in a multidisciplinary team meeting in order to rapidly start these patients on the appropriate therapeutic regimen. When a non-ophthalmologist encounters a patient with a systemic disease with nonspecific eye symptoms, it is important to refer this patient to an ophthalmologist to exclude possible concurrent active uveitis.

## 5. Conclusions

Since many disorders can cause uveitis, patients with uveitis are often managed by a team of medical subspecialists. Rapidly rendering a uveitis diagnosis is critical to prevent irreversible damage. The underlying cause of uveitis dictates the therapeutic approach. The management of severe cases of uveitis should be coordinated by an experienced multidisciplinary team comprising both ophthalmologists and other medical specialists.

Biologicals such as TNF-α inhibitors and IFN-α have expanded the treatment options for chronic non-infectious uveitis in the last decades, albeit the financial consequences of this development remain to be elucidated. Nevertheless, these drugs have been shown to translate to considerable improvements in the treatment of certain uveitis patients.
